# The Spatial and Cell-Type Distribution of SARS-CoV-2 Receptor ACE2 in the Human and Mouse Brains

**DOI:** 10.3389/fneur.2020.573095

**Published:** 2021-01-20

**Authors:** Rongrong Chen, Keer Wang, Jie Yu, Derek Howard, Leon French, Zhong Chen, Chengping Wen, Zhenghao Xu

**Affiliations:** ^1^Institute of Traditional Chinese Medicine Clinical Basic Medicine, School of Basic Medical Science, Zhejiang Chinese Medical University, Hangzhou, China; ^2^Krembil Centre for Neuroinformatics, Centre for Addiction and Mental Health, Toronto, ON, Canada; ^3^Zhejiang Chinese Medical University, Hangzhou, China; ^4^Key Laboratory of Medical Neurobiology of National Health Commission and Chinese Academy of Medical Sciences, Institute of Pharmacology and Toxicology, College of Pharmaceutical Sciences, Zhejiang University, Hangzhou, China

**Keywords:** angiotensin-converting enzyme 2, ACE2, brain, SARS-coronavirus 2, COVID-19

## Abstract

By engaging angiotensin-converting enzyme 2 (ACE2 or Ace2), the novel pathogenic severe acute respiratory syndrome coronavirus 2 (SARS-CoV-2) invades host cells and affects many organs, including the brain. However, the distribution of ACE2 in the brain is still obscure. Here, we investigated the ACE2 expression in the brain by analyzing data from publicly available brain transcriptome databases. According to our spatial distribution analysis, ACE2 was relatively highly expressed in some brain locations, such as the choroid plexus and paraventricular nuclei of the thalamus. According to cell-type distribution analysis, nuclear expression of ACE2 was found in many neurons (both excitatory and inhibitory neurons) and some non-neuron cells (mainly astrocytes, oligodendrocytes, and endothelial cells) in the human middle temporal gyrus and posterior cingulate cortex. A few ACE2-expressing nuclei were found in a hippocampal dataset, and none were detected in the prefrontal cortex. Except for the additional high expression of Ace2 in the olfactory bulb areas for spatial distribution as well as in the pericytes and endothelial cells for cell-type distribution, the distribution of Ace2 in the mouse brain was similar to that in the human brain. Thus, our results reveal an outline of ACE2/Ace2 distribution in the human and mouse brains, which indicates that the brain infection of SARS-CoV-2 may be capable of inducing central nervous system symptoms in coronavirus disease 2019 (COVID-19) patients. Potential species differences should be considered when using mouse models to study the neurological effects of SARS-CoV-2 infection.

## Introduction

Since December 2019, much attention has focused on the novel severe acute respiratory syndrome coronavirus 2 (SARS-CoV-2) and the related coronavirus disease 2019 (COVID-19) pandemic, which is rapidly spreading around the world and results in a global health emergency (1). In addition to atypical pneumonia, the central nervous system (CNS) symptoms of COVID-19 patients have been observed in the clinic (2). According to a recent retrospective case series study, 53 out of 214 (24.8%) COVID-19 patients had CNS symptoms, including dizziness, headache, impaired consciousness, acute cerebrovascular disease, ataxia, and epilepsy (3). More importantly, it has been found that SARS-CoV, a previously reported similar coronavirus, spreads into the brain after it was cleared from the lung in mice, which could be more concealment than that in the lung (4). Recently, Puelles et al. found that SARS-CoV-2 has an organotropism beyond the respiratory tract, including the kidneys, liver, heart, and brain (5). Thus, it is necessary and urgent to study the CNS infection of SARS-CoV-2.

Angiotensin-converting enzyme 2 (ACE2 or Ace2) has been identified as a key entry receptor for novel pathogenic SARS-CoV-2 invasion, similar to previous SARS-CoV (6). By binding of the spike protein to ACE2, SARS-CoV-2 and SARS-CoV could invade host cells in human organs (7, 8). However, the distribution of ACE2 in the brain is still obscure and even inconsistent. In 2004, Hamming et al. found that ACE2 may be expressed only in the endothelium and vascular smooth muscle cells in the human brain tissue (9). However, some recent studies found that the human endothelial cells expressed low or undetectable levels of ACE2 (10, 11). On the other hand, a previous study has reported that Ace2 could be expressed in the mouse neuron cells, which may contribute to the development of hypertension (12); however, in another neurocytometry study, Ace2 is a potential marker for non-neurons in the zinc-fixed mouse brain cortical section (13). Thus, further clarifying the brain tissue distribution of ACE2 may help to bring to light the CNS infection of the novel SARS-CoV-2 and previous SARS-CoV.

Here, we investigated the distribution of ACE2 in the brain by analyzing publicly available brain transcriptome databases. These databases, such as those produced by the Allen Institute for Brain Science, offer an extremely valuable source of genomic data, whose processing and interpretation may facilitate translational research (14, 15). We revealed an uneven spatial and cell-type distribution of ACE2 in the human and mouse brains.

## Methods

### Brain Transcriptome Databases

As listed in Table 1, 10 publicly available brain transcriptome databases that can be accessed without specialized computational expertise were used. All these databases offer extremely valuable sources of genomic data freely for academic and other non-commercial purposes. Except for the Single Cell Portal database (https://singlecell.broadinstitute.org), some of these databases have been also introduced in a recent review study (27). All databases and datasets were appropriately used and cited according to their citation policy, license, or terms of use.

**Table 1:** The database used for the current study.

**Analysis**	**Web Interface**	**References**	**Species**	**Brain area**	**Method**
Spatio-temporal	http://hbatlas.org	(16)	Human	Multi	Microarray
Spatial	http://human.brain-map.org	(17)	Human	Multi	Microarray
Spatial	https://www.gtexportal.org	(18)	Human	Multi	RNA-seq
Spatial	https://mouse.brain-map.org/	(19)	Mouse	Multi	Microarray
Spatial and cell type	https://hipposeq.janelia.org	(20)	Mouse	Hippocampus	RNA-seq
Cell type	http://mousebrain.org	(21)	Mouse	Multi	RNA-seq
Cell type	https://www.brainrnaseq.org/	(22, 23)	Mouse and human	Multi	RNA-seq
Single cell	https://singlecell.broadinstitute.org	Not available	Many	Multi	RNA-seq
Single cell	https://celltypes.brain-map.org/rnaseq/	(24)	Human	Cortex	RNA-seq
Single cell	http://betsholtzlab.org/VascularSingleCells/database.html	(25, 26)	Mouse	Blood vessels	scRNA-seq

### Analysis of the Spatial Distribution of ACE2 in the Human Brain

Three databases, including Allen Human Brain Atlas database (http://human.brain-map.org), Human Brain Transcriptome database (https://hbatlas.org), and GTEx Portal database (https://www.gtexportal.org), were used to analyze the spatial distribution of ACE2 in the human brain.

### Cell-Type Distribution of ACE2 in the Human Brain

Three single-cell sequencing databases, including Single Cell Portal database (https://singlecell.broadinstitute.org, single-cell sequencing), Allen Cell Types database (http://celltypes.brain-map.org, single-cell sequencing), and Brain RNA-Seq database from Barres labs (https://www.brainrnaseq.org/, RNA-seq of cell types isolated from the human brain), were used. The summary of the included datasets for the human brain is shown in Table 2.

**Table 2:** Summary of cell-type or single-cell sequencing databases for the human and mouse brains.

**Brain Area**	**References**	**Method**	**Nuclei**	**Species n (age)**	**Source**
Middle temporal gyrus	(24)	SMART-Seq	15,928	Human *n* = 8 (24–66)	https://celltypes.brain-map.org/rnaseq/human/mtg
Posterior cingulate cortex	(28)	Multiplexing snRNA-seq	9,923	Human *n* = 20 (>65)	https://singlecell.broadinstitute.org/single_cell/study/SCP371
Prefrontal cortex and hippocampus	(29)	DroNc-Seq	19,550	Human *n* = 5 (40–65)	https://singlecell.broadinstitute.org/single_cell/study/SCP90/
Temporal lobe cortex and hippocampus	(22, 23)	RNA-seq of cell types isolated from the human brain	NA	Human *n* = 22 (8–65)	https://www.brainrnaseq.org/
Whole cortex	(30)	10 × Chromium, SMART-seq2, DroNc-seq and sci-RNA-seq	13,783	Mouse (1 month old)	https://singlecell.broadinstitute.org/single_cell/study/SCP425/
SNr, SNc, VTA	U19—Huang BICCN data (1U19MH114821-01)^a^	Unclear	13,861	Mouse (unclear)	https://singlecell.broadinstitute.org/single_cell/study/SCP478
Cerebellum	(31)	High-throughput single-nucleus RNA-seq	611,034 (10,000 used)	Mouse 2 female, 4 male (60 days)	https://singlecell.broadinstitute.org/single_cell/study/SCP795
Prefrontal cortex and hippocampus	(29)	DroNc-Seq	29,543	Mouse 4 (adult)	https://singlecell.broadinstitute.org/single_cell/study/SCP60
Hippocampus	(20)	Div-Seq	1,367	Mouse unclear	https://singlecell.broadinstitute.org/single_cell/study/SCP1
Multiple areas (265 clusters of cells)	(21)	Cell-type RNA-seq	509,876 (160,796 used)	Mouse male and female (12–56 days old)	http://mousebrain.org
Vascular and vessel-associated cells (15 of cluster cells)	(25, 26)	scRNA-seq	3,186	Mouse (adult)	http://betsholtzlab.org/VascularSingleCells/database.html

a *This dataset is provided by Huang et al. (https://biccn.org/teams/u19-huang) and from the BRAIN Initiative Cell Census Network (BICCN, https://biccn.org/). Multiplexing snRNA-seq: nuclei multiplexing with barcoded antibodies for single-nucleus genomics; Smart-Seq: switching mechanism at 5′ end of the RNA transcript; DroNc-Seq: deciphering cell types in the human archived brain tissues by massively-parallel single-nucleus RNA-seq; sci-RNA-seq: single-cell combinatorial-indexing RNA-sequencing analysis; SNr: substantia nigra pars reticulate; SNc: substantia nigra pars compacta; VTA: ventral tegmental area*.

### Spatial and Cell-Type Distribution of Ace2 in the Mouse Brain

Allen Mouse Brain Atlas database (http://mouse.brain-map.org) was used to analyze the general spatial distribution of Ace2 in the mouse brain. The hippocampus RNA-seq Atlas database (https://hipposeq.janelia.org/), Single Cell Portal database, Mouse Brain Atlas database (http://mousebrain.org), Brain RNA-Seq database from Barres lab (https://www.brainrnaseq.org/), and Brain vascular single-cell database from Betsholtz lab were used for cell-type distribution of Ace2 in the mouse brain. The summary of the used five single-cell sequencing datasets is also shown in Table 2.

### Data Processing and Statistical Analysis

Datasets were independently searched and analyzed by two authors (RC and JY), and any disagreements were discussed and resolved by consensus with the corresponding author (ZX). The data from the Allen Human Brain Atlas database and Allen Cell Types database were exported to Microsoft Excel 2017 and GraphPad Prism 6.0 for further analysis. ACE2 expression data from the Allen Human and Mouse Brain Atlas databases were imported to Brain Explorer 2.0 software to get visualization. The data from the other databases were analyzed online. Where applicable, data are expressed as median or mean. Interquartile range (IQR), 95% CI, range, and/or all sample points were also provided if possible.

Positive of expression criterion: (1) to minimize false positive of expression as previous studies (32, 33), a gene with calculate counts per million (CPM), transcripts per million (TPM), unique molecular identifier (UMI) count, or fragments per kilobase million (FPKM) >1 were considered to be positive, which also is the same as log10 (CPM), log10 (TPM), or log10 (UMI) ≥0 as well as log10 (CPM + 1), log10 (TPM + 1), and log10 (UMI + 1) ≥0.3; (2) besides, as a z score being >2 corresponds to a *p*-value <0.05, a z score of ACE2 expression >2 was considered as high ACE2 expression in the Allen Human Brain Atlas database.

## Results

### The General Expression of ACE2 in the Human Brain

**Figure 1:**
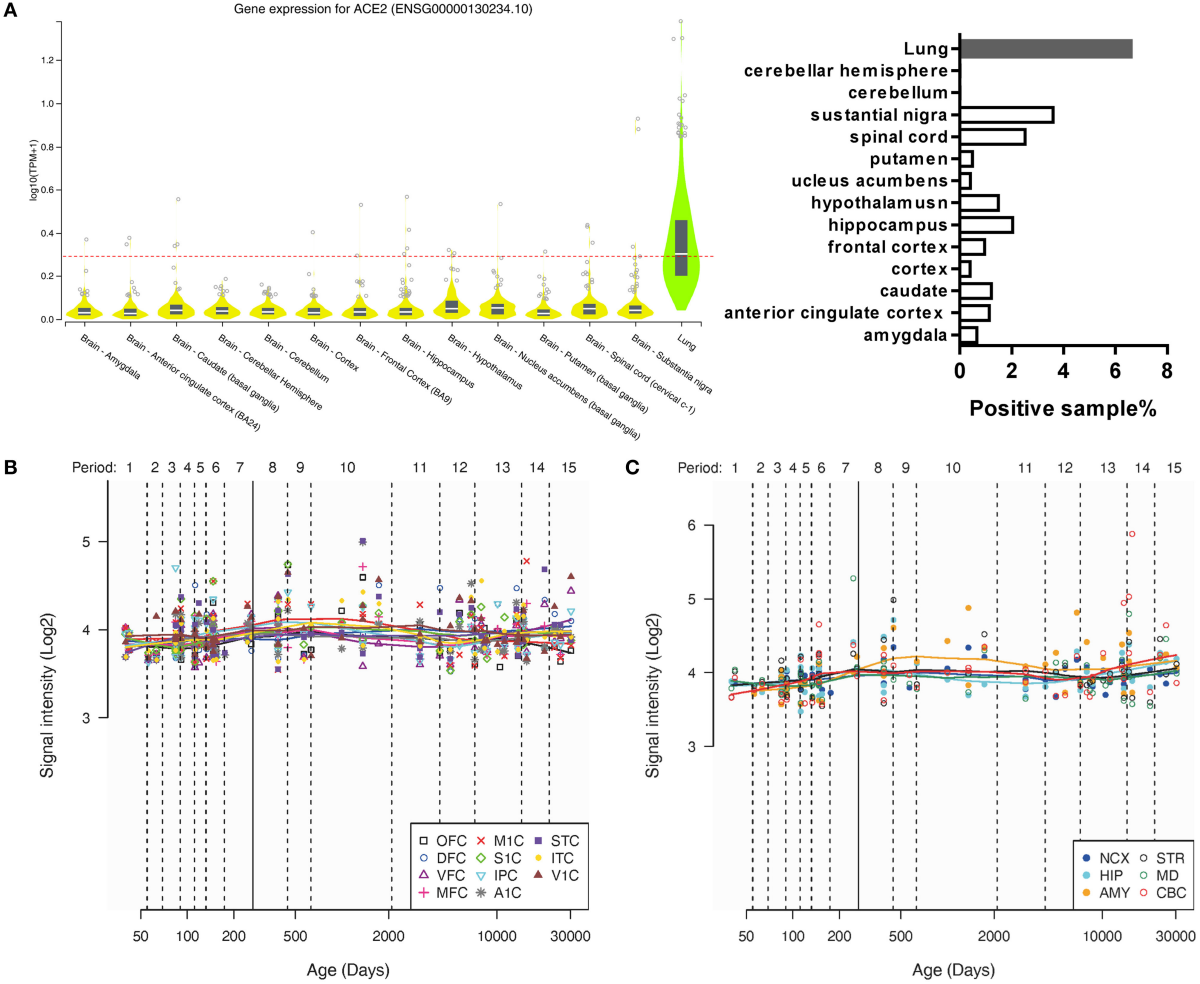
The general expression of ACE2 in different human brain areas. **(A)** The expression of ACE2 in different human brain areas and the lung according to the GTEx Portal database (18). The dotted line means log10(CPM + 1) = 0.3, which means a threshold of the positive sample (>0.3 could be positive). **(B,C)** Change of intensity of ACE2 expression with age in different human brain areas according to the Human Brain Transcriptome database. Data are expressed as median, interquartile range (IQR), and all sample points in **(A)**, whereas data are expressed as mean and all sample points in **(B,C)**.

According to the GTEx Portal database, the general expression of ACE2 was extremely low but not none in the brain according to our positive of expression criterion (Figure 1A). Excepted for the lung (38 out of 578, positive rate of 6.57%), the positive samples were also found in the amygdala (1 out of 152, 0.65%), anterior cingulate cortex (2 out of 176, 1.14%), caudate (3 out of 246, 1.22%), cortex (1 out of 255, 0.39%), frontal cortex (2 out of 209, 0.96%), hippocampus (4 out of 197, 2.03%), hypothalamus (3 out of 202, 1.49%), nucleus accumbens (NAc; 1 out of 246, 0.41%), putamen (1 out of 205, 0.48%), spinal cord (cervical c-1, 4 out of 159, 2.52%), and substantial nigra (5 out of 139, 3.60%), but none in the cerebellum (0 out of 241) and cerebellar hemisphere (0 out of 215).

In addition, according to the Human Brain Transcriptome database, the general expression of ACE2 was also similar among the cortex and other brain regions, which may not change a lot with age (Figures 1B,C).

### Two-Spatial Distribution of ACE2 in the Human Brain

**Figure 2:**
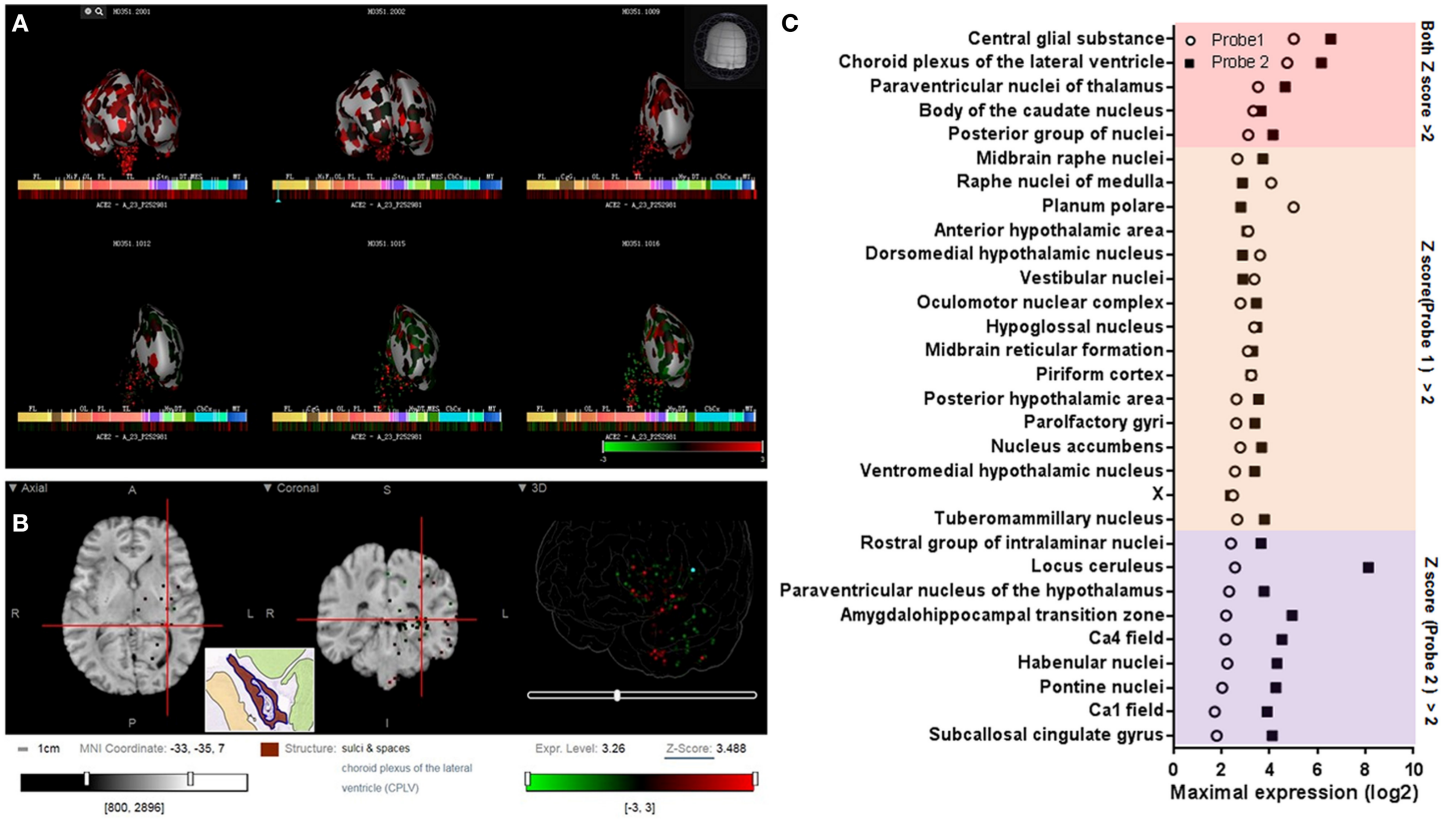
Spatial distribution of ACE2 in the human brain according to the Allen Human Brain Atlas. **(A)** 3D view of the expression of ACE2 in different human brain areas based on the data of probe A_23_P252981 (probe 1). **(B)** Planar view of the expression of ACE2 in the choroid plexus of the lateral ventricle. The inset shows a sampled area of the choroid plexus of the lateral ventricle (the dark area in the inset). **(C)** Log2 intensity of ACE2 expression in the 30 brain areas with a z score >2.0 in at least one probe dataset (probe 1: A_23_P252981; probe 2: CUST_16267_PI416261804). All data were generated from the Allen Human Brain Atlas (http://human.brain-map.org/microarray/search). Images in **(A,B)** were directly from the Allen Human Brain Atlas (© 2010 Allen Institute for Brain Science, Allen Human Brain Atlas, available from: http://human.brain-map.org).

By providing slice images, the Allen Human Brain Atlas may prove a more detailed spatial distribution of ACE2 in the human brain than the GTEx Portal database and Human Brain Transcriptome database. Two microarray datasets using the different probes of ACE2 (A_23_P252981, CUST_16267_PI416261804) were collected from the Allen Human Brain Atlas database (http://human.brain-map.org/microarray/search). To provide a relatively systematic analysis of the Allen Human Brain Atlas database, we analyzed the data as both separated datasets and pooled them together. By analyzing the z score and intensity of ACE2 expression as two separated datasets, we found that 6 brain areas had high ACE2 expression (with a maximal z score of ACE2 expression >2.0) in both probe datasets, 15 brain areas had high ACE2 expression only in probe 1 dataset, and 9 brain areas had high ACE2 expression only in probe 2 dataset (Figures 2A,B).

**Figure 3:**
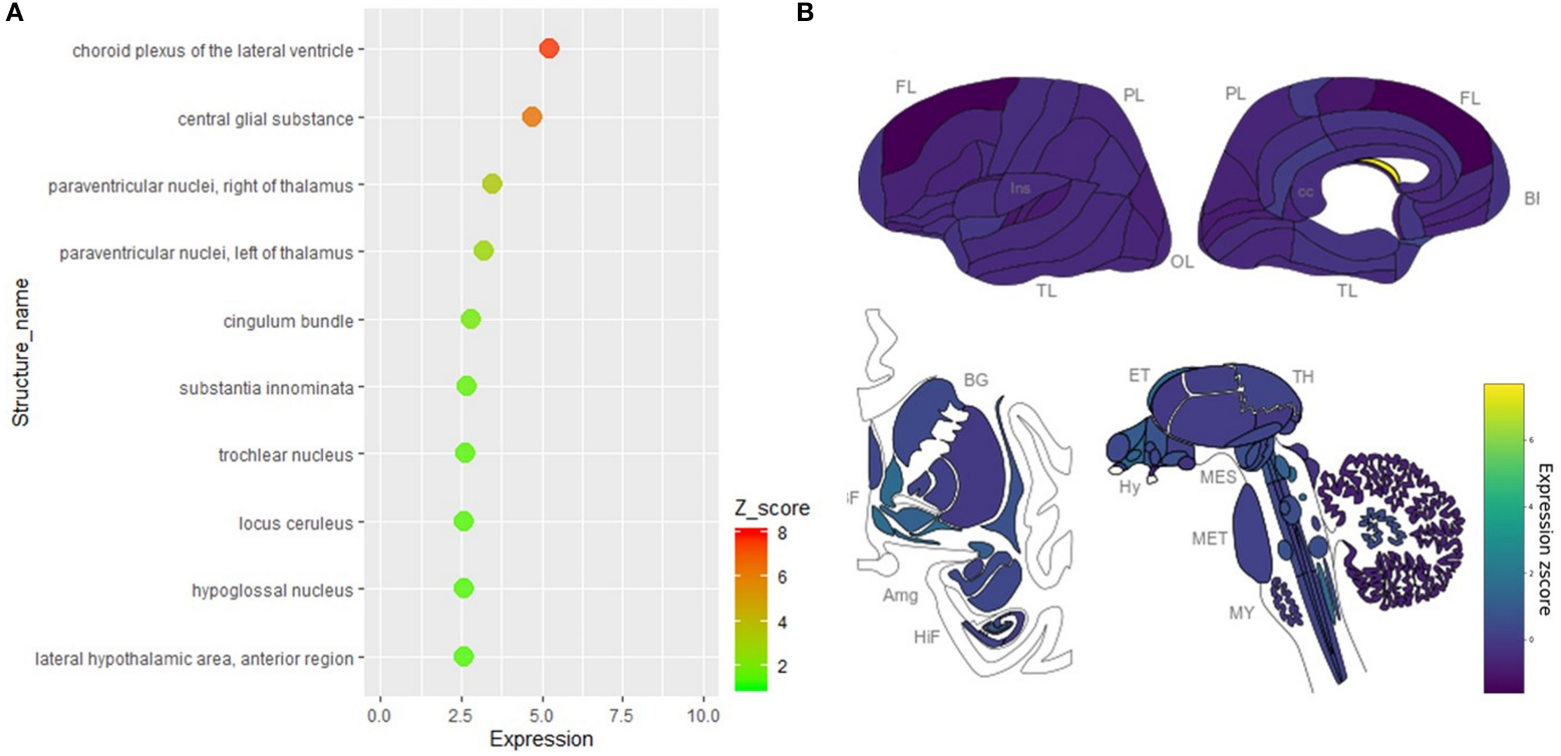
Spatial distribution of ACE2 in the human brain according to the pooled data of the Allen Human Brain Atlas. **(A)** The expression intensity and z score of ACE2 in the top 10 brain areas of the pooled expression data of the two probes. **(B)** The distribution view of ACE2 expression in the human brain according to the pooled expression data of the two probes. Original data are available from the Allen Human Brain Atlas (http://human.brain-map.org/microarray/search). Brain region abbreviations: FL, frontal lobe; PL, parietal lobe; TL, temporal lobe; OL, occipital lobe; BF, basal forebrain; BG, basal ganglia; AmG, amygdala; HiF, hippocampal formation; EP, epithalamus; TH, thalamus; Hy, hypothalamus; MES, mesencephalon; MET, metencephalon; MY, myelencephalon.

To further visualize the spatial distribution of ACE2 expression and minimize the influences of the use of two different probes (such as the difference of detective sensitivity), we pooled the expression value of the two datasets and found 23 brain areas with a z score >1.0 and four of them with a z score >2.0 (Figure 3A). The spatial distribution of ACE2 expression in the human brain according to the pooled expression data is shown in Figure 3B.

### Cell-Type Distribution of ACE2 in the Human Brain

We further collected and analyzed single-cell sequencing data, which may provide all mRNAs present in every single cell of the tested brain tissue. By analyzing the single-cell sequencing data of the human middle temporal gyrus (https://celltypes.brain-map.org/rnaseq/human/mtg), human posterior cingulate cortex (https://singlecell.broadinstitute.org/single_cell/study/SCP371), and archived human prefrontal cortex (PFC) and hippocampus samples (https://singlecell.broadinstitute.org/single_cell/study/SCP90/), the expression of ACE2 is relatively high in the human middle temporal gyrus (a total of 2.00% of the ACE2 positive cells, 309 out of 15,603) and posterior cingulate cortex (a total of 1.38% of the ACE2 positive cells, 133 out of 9,635) samples, but it is very low in the archived human PFC (no ACE2 positive cells) and hippocampus samples (a total of 0.21% of the ACE2 positive cells, 2 out of 9,530). Besides, according to the Brain RNA-Seq database from Barres lab, we also found no expression of ACE2 in all kinds of cell subtypes that were isolated from the human anterior temporal lobe cortex and hippocampus (Supplementary Figure 1).

**Figure 4:**
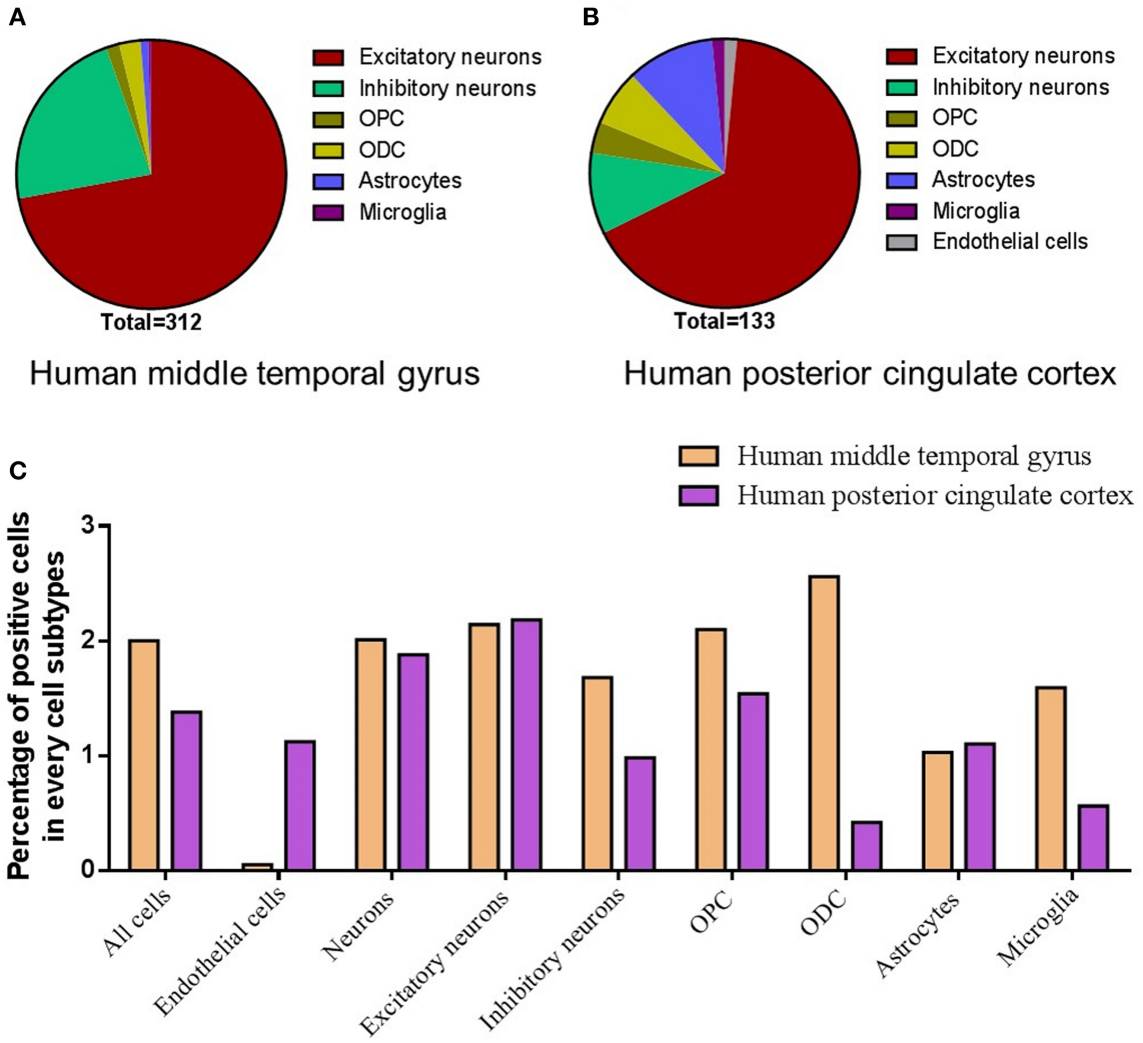
Cell-type distribution of ACE2 in the human brain. **(A)** The cell-type proportion of the total positive cells in the human middle temporal gyrus. **(B)** The cell-type proportion of the total positive cells in the human posterior cingulate cortex. **(C)** The percentage of positive cells in different cell subtypes in the human middle temporal gyrus and posterior cingulate cortex. Original data are from https://celltypes.brain-map.org/rnaseq/human/mtg and https://singlecell.broadinstitute.org.

For the cell types, most of the ACE2 positive cells were neurons in both the human middle temporal gyrus and the posterior cingulate cortex, especially excitatory neurons (72.1% for the middle temporal gyrus and 66.1% for the posterior cingulate cortex, Figures 4A,B) and interneurons (22.4% for the middle temporal gyrus and 9.8% for the posterior cingulate cortex, Figures 4A,B). The ACE2 positive percentage of excitatory neurons was also the highest among cell types (2.14% for the middle temporal gyrus and 2.18% for the posterior cingulate cortex, Figure 4C). The details of the cell-type distribution of ACE2 in the human brain are shown in Supplementary Figures 2, 3.

### Spatial and Cell-Type Distribution of Ace2 in the Mouse Brain

**Figure 5:**
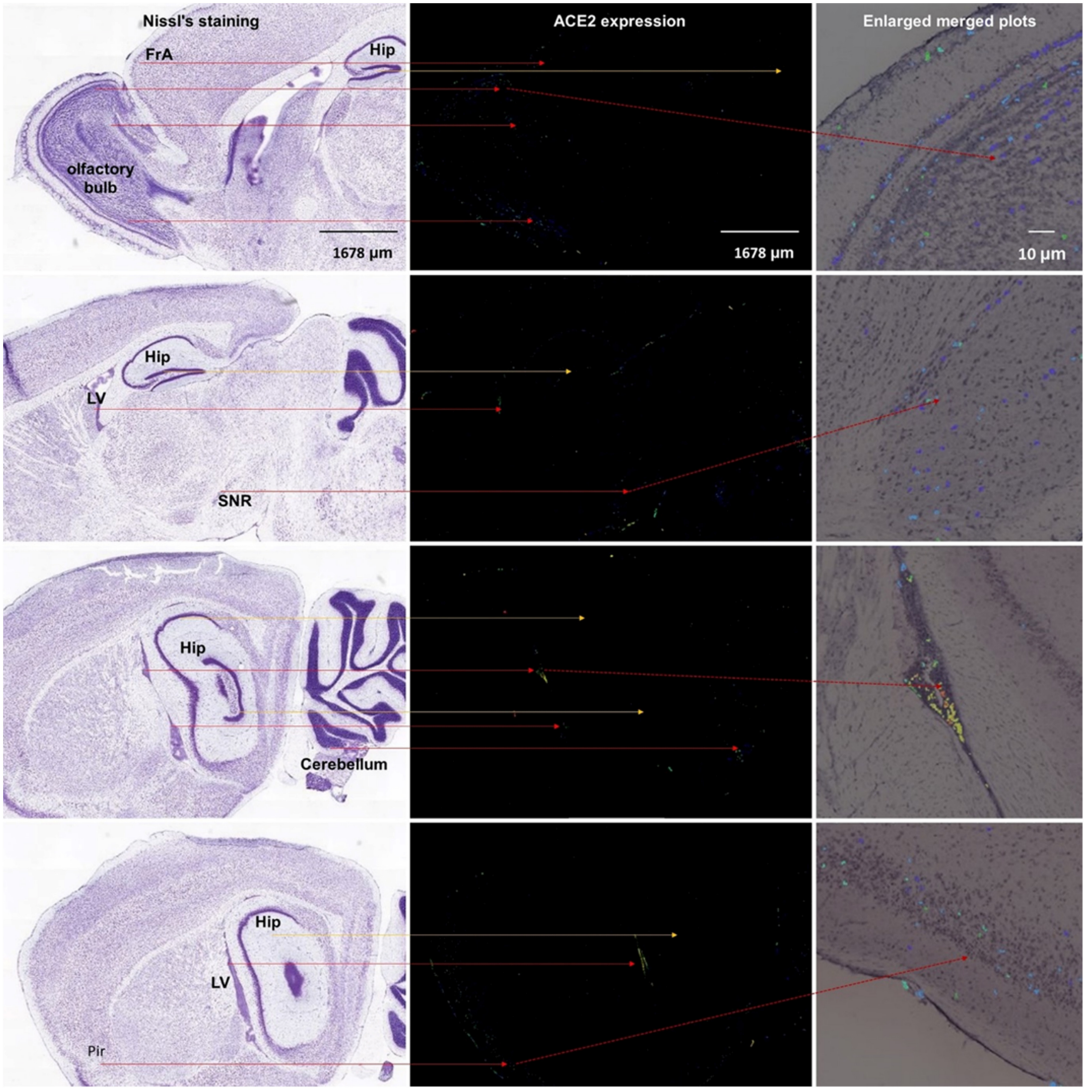
Spatial distribution of ACE2 in the mouse brain. The left column means the Nissl staining of mouse brain slice, the middle column means the ACE2 expression by antisense, and the right column means the enlarged merged plots from the Brain Explorer 2.0 software. Hip, hippocampus; LV, lateral ventricle; SNR, substantia nigra pars reticulate; Pir, piriform cortex. All images are from the Allen Mouse Brain Atlas (© 2004 Allen Institute for Brain Science, Allen Mouse Brain Atlas, available from https://mouse.brain-map.org/).

As shown in Figure 5, we additionally analyzed the spatial distribution of Ace2 in the mouse brain based on the Allen Mouse Brain Atlas (http://mouse.brain-map.org/gene/show/45849). Similar to the human brain, we found that the Ace2 expression is relatively high in the choroid plexus of the lateral ventricle, substantia nigra pars reticulata (SNr), and some cortical areas (such as the piriform cortex). We additionally found that the ACE2 expression is also relatively high in the olfactory bulb, whereas it was very low in the hippocampal areas.

We further analyze the single-cell sequencing data of the multiple mouse cortex (https://singlecell.broadinstitute.org/single_cell/study/SCP425/); archived mouse brain samples (https://singlecell.broadinstitute.org/single_cell/study/SCP60); mouse hippocampus (https://singlecell.broadinstitute.org/single_cell/study/SCP1); substantia nigra pars reticulate (SNr), substantia nigra pars compacta (SNc), and ventral tegmental area (VTA) areas (https://singlecell.broadinstitute.org/single_cell/study/SCP478); and cerebellum (https://singlecell.broadinstitute.org/single_cell/study/SCP795). Similar to human data, the expression of Ace2 is relatively high in the multiple mouse cortex samples (a total of 0.84% of the Ace2 positive cells, 84 out of 10,000), but it is relatively low in the mixed mouse PFC and hippocampus samples (a total of 0.28% of the Ace2 positive cells, 38 out of 13,313) and the mouse hippocampus samples (a total of 0.1% of the Ace2 positive cells, 2 out of 1,188). Besides, the expression of Ace2 is also found in 0.68% of the cell in the mixed SNr, SNc, and VTA areas as well as 0.52% in the cerebellum.

**Figure 6:**
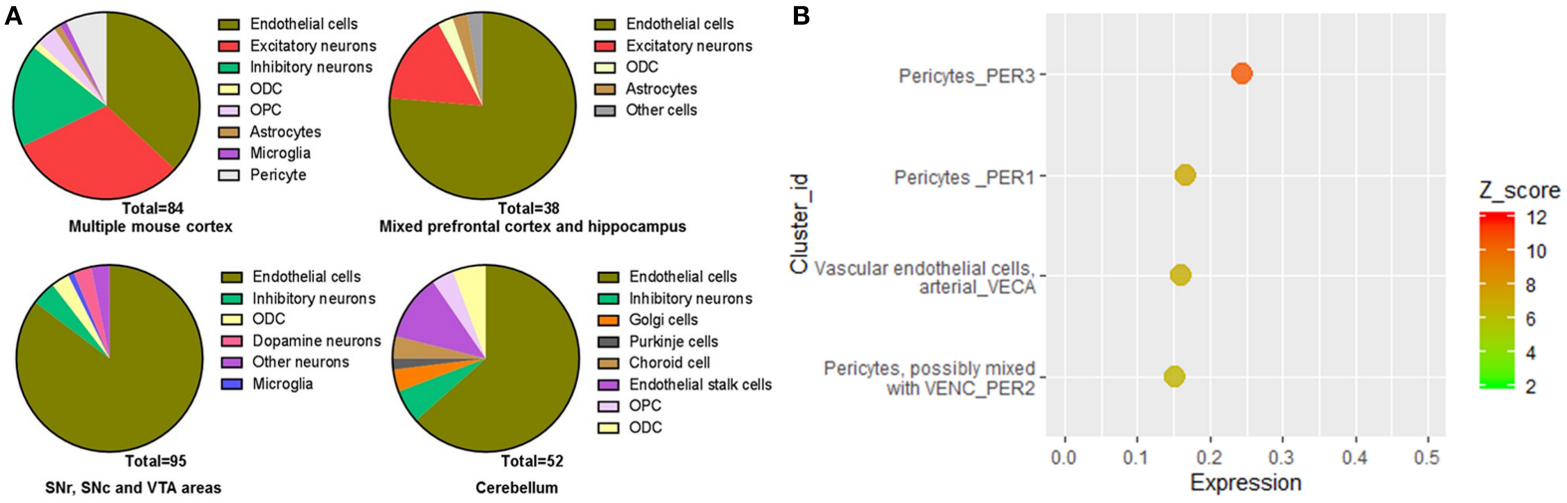
Cell-type distribution of Ace2 in the mice brain. **(A)** Cell-type distribution of Ace2 according to the Single Cell Portal database (https://singlecell.broadinstitute.org/). **(B)** Top cell-type cluster expression of Ace2 according to the Mouse Brain Atlas database (http://mousebrain.org).

For the cell-type distribution, different from human brain data, the Ace2 positive rate of the endothelial cells was the highest among different cell types in the mouse brain, including the multiple mouse cortex; mixed PFC and hippocampus; mixed SNr, SNc, and VTA areas; and cerebellum (Figure 6A). Similar to human brain data, the Ace2 positive rate of the neurons was also very high among different cell types in the mouse brain, including the multiple cortex (48.8%); mixed PFC and hippocampus (15%); mixed SNr, SNc, and VTA areas (10.5%); and cerebellum (7.69%) (Figure 6A). The details of the cell-type distribution of Ace2 in the mouse brain are shown in Supplementary Figure 4. Similarly, in a comprehensive atlas of the mouse nervous system, of the 265 cell clusters, 4 had an expression z score >2 (3 pericyte clusters and 1 cluster of arterial endothelial cells, Figure 6B). According to the Brain RNA-Seq database from Barres lab, Ace2 was also highly expressed in the endothelial cells in the mouse brain (Supplementary Figure 1). Besides, according to the vascular single-cell transcriptions database from Betsholtz labs (Supplementary Figure 5), we further found: (1) ACE2 was only expressed in one kind of the endothelial cells in the brain vascular and (2) the expression level of ACE2 in the endothelial cells was much lower than that in the pericytes, smooth muscle cells, vascular fibroblast-like cells, and brain vascular.

As the inconsistent results were found in the hippocampus based on the single-cell sequencing, we additionally analyzed the expression of Ace2 in a cell-type sequencing database of the mouse hippocampus, the Hippocampus RNA-seq Atlas database (https://hipposeq.janelia.org). As shown in Supplementary Figure 6, (1) Ace2 expression was only found in the intermediate and ventral areas of the hippocampus with a mean FPKM <1; (2) Ace2 expression was found in the ventral pyramidal cells but not in the dorsal CA1 pyramidal cells, dorsal CA3 pyramidal cells, dorsal CA2 pyramidal cells, dorsal DG granule cells, dorsal DG mossy cells, ventral CA3 pyramidal cells, and ventral DG granule cells; (3) Ace2 expression was not found in parvalbumin (PV)-positive or somatostatin (SST)-positive interneurons in the hippocampus; and (4) Ace2 expression was not found in any neurons that project to the postsubiculum, NAc, or amygdala.

## Discussion

ACE2 is an important entry receptor for SARS-CoV-2 and SARS-CoV infecting host organs (6). Though the infection of SARS-COV in the brain was reported in the past (34, 35), the distribution of ACE2 in the brain is still unclear. Here, we mainly found: (1) the expression of ACE2 was relatively high in several specific brain areas in human; (2) the expression of ACE2 was mainly located in many neurons (both excitatory and inhibitory neurons) in the human middle temporal gyrus and posterior cingulate cortex, but it was undetectable in the human PFC and very low in the human hippocampus; and (3) except for the additional expression of Ace2 in the olfactory bulb areas for spatial distribution and the pericytes and endothelial cells for cell-type distribution analysis, the main distribution map of Ace2 in the mouse brain was similar to that in human. Thus, our results revealed an outline of ACE2 or Ace2 distribution in the human and mouse brains, which supports the hypothesis that SARS-CoV-2 is capable to infect the brain and lead to CNS symptoms in COVID-19 patients (36). Our results indicate a heterogeneous distribution of ACE2 in different brain areas and cell types, which should be considered in further related studies.

SARS-CoV-2 shares a 77.2% amino acid identity, 72.8% sequence identity, and high structural similarity to previous SARS-CoV (37, 38). Similar to SARS-CoV, experimental affinity measurements show a high affinity of the receptor-binding domain of SARS-CoV-2 and ACE2 (37, 39). According to the GTEx Portal database, we found: (1) the total expression in the brain was much lower than that in the lung and (2) however, ACE2 positive samples were found in many brain areas, especially in the substantial nigra that the ACE2 positive sample rate was almost comparable to that in the lung. According to the Human Brain Transcriptome database, the total expression of ACE2 seems not to change with age. Moreover, according to the Allen Brain Atlas, six brain areas had relatively high expression of ACE2, especially four brain areas had high expression of ACE2 in the pooled data. Some of these high ACE2 expression brain nuclei are very important for normal brain functions, such as the paraventricular nuclei of the thalamus (involved in the control of wakefulness, feeding, appetitive motivation, drug addiction, regulation of stress and negative emotional behavior, and epilepsy) (40, 41), the raphe nuclei (the main serotoninergic nuclei in the brain) (42), and tuberomammillary nucleus (the main histaminergic nuclei in the brain) (43). Therefore, our results highlight the importance of spatial distribution rather than the overall expression of ACE2 in the brain. These results may provide some clues for further study on the brain infection of SARS-CoV-2 in COVID-19 patients and suggest that SARS-CoV-2 may result in serious CNS symptoms in COVID-19 patients (if it would infect these important brain areas by binding ACE2). Brain imaging and long-term follow-up may be required to confirm the possibility of SARS-CoV-2 brain infection and the following brain disorders in COVID-19 patients.

The routes or pathways for SARS-CoV and novel SARS-CoV-2 entering the brain are still unclear. According to experiments in mice transgenic for human ACE2, intranasal administration of SARS-CoV may enter the brain through the olfactory nerves (44). Consistent with this, we found that the expression of ACE2 in the olfactory bulb is higher than that in most other cortexes (Figure 3). In the human brain, we also found that the piriform cortex, a brain area directly connected with the olfactory bulb, had high ACE2 expression. Though no ACE2 expression data of the olfactory bulb in humans were available, our results indirectly support the hypothesis that SARS-CoV-2 might enter the human brain through the olfactory nerves. Recently, Meinhardt et al. found the olfactory transmucosal SARS-CoV-2 invasion as a port of CNS entry in individuals with COVID-19, which supports our finding (45).

On the other hand, we additionally found high ACE2 expression (z score >5) in the central glial substance and choroid plexus of the lateral ventricle in the human brain. Relatively high expression of ACE2 in the choroid plexus of the lateral ventricle was also found in the mouse brain in the current study. The central glial substance refers to an area of gray matter surrounding the central canal, which carries the cerebrospinal fluid (CSF) and helps to transport nutrients to the spinal cord (46). Besides, the choroid plexus of the ventricles is an important brain area for the generation of the CSF (47), the main location of the blood–CSF barrier (48), and serves as a crucial gateway for immune cells entering the brain (49). Recently, SARS-CoV-2 has also been found in CSF samples from a 56-year-old COVID-19 patient by genetic sequencing in China (http://www.ecns.cn/news/society/2020-03-05/detail-ifzuhesu4119860.shtml). SARS-CoV-2 may also infect the brain of a 24-year-old male patient (33). Thus, our results suggest that the high expression of ACE2 in the central glial substance and ventricles may provide another potential pathway for SARS-CoV-2 or SARS-CoV entering the CSF and/or spreading around the brain. This idea has been further confirmed by two recent studies, which found that SARS-CoV-2 infects the brain choroid plexus in human brain organoids (50, 51).

Single-nucleus RNA-seq provides a high resolution of cellular gene expression of each cell (52). According to the single-nucleus RNA-seq data, we further found that ACE2 was located in many neurons (especially excitatory neurons) and some non-neuron cells [especially astrocytes and oligodendrocytes (ODCs)] in both the posterior cingulate cortex and the middle temporal gyrus. The highest number of ACE2 positive cells was in the excitatory neurons, which may be projection neurons that make up many important brain networks. For example, excitatory neurons in the posterior cingulate cortex may project dense connections to the hippocampal formation and parahippocampal cortex, which are related to emotion and memory (53). On the other hand, the percentage of positive cells in some inhibitory neuron subtypes was comparable compared with excitatory neurons, though the total positive cell number of inhibitory neurons is lower than excitatory neurons. Inhibitory neurons are crucial for normal brain function (54). For example, the neurons in the SNr are mainly inhibitory gamma-aminobutyric acid (GABAergic) neurons, which is one important note in the neural circuits that contribute to epilepsy (41). Besides, we also found that some dopaminergic neurons and cerebellar cells in the mouse brain are also ACE2 positive. Thus, our results may help to explain the previous finding that SARS-CoV particles are mainly located in the neurons in the brain samples from SARS patients (34) and suggest that SARS-CoV-2 may also invade many neurons in the human brain and hence contribute to the CNS symptoms in COVID-19 patients.

In addition, single-nucleus RNA-seq showed that the expression of ACE2 was very low in both the endothelial cells and the pericytes from the human brain, whereas the expression of Ace2 was high in the endothelial cells and the pericytes in the mouse brain. According to RNA-seq of isolated subtype cells from the human and mouse brains, we also found that the expression of Ace2 was high in the mouse endothelial cells, but that of ACE2 was none in the human endothelial cells. Of note, previous microarray data show ACE2 expressed in the human endothelium (9), which is mainly a single layer of squamous endothelial cells. One possible reason for these differences might be that most blood vessels were excluded from the tested human brain tissues, whereas they were hard to be removed and left in the mouse brain tissues. However, some recent studies found that the human vascular endothelial cells do not express ACE2 or express relatively low levels of ACE2 (10, 11). Besides, according to the vascular single-cell transcriptions database from Betsholtz labs, the expression of Ace2 only found in one subtype of the vascular endothelial cells in the mouse brain and its level was also very low. On the contrary, the expression of Ace2 in the pericytes is very high in the mouse brain according to the database from Betsholtz labs. Thus, these results taken together suggest: (1) blood vessels may not be the main source of ACE2 expressed endothelial cells in mice; (2) the heterogeneity of ACE2 or Ace2 distribution in the subtypes of the endothelial cells or pericytes may exist in both the human and mouse brains; and (3) such a potential difference between humans and mice should be noted when using mouse models for the SARS-CoV-2 study. Further studies are need to confirm the ACE2 distribution in the specific types of the endothelial cells or pericytes (such as these nonvascular endothelial cells or pericytes in the cerebral lymphatic or ventricles), which may help SARS-CoV-2 to enter the brain and contribute to the cerebrovascular events in COVID-19 patients (55–57).

## Conclusions

**Figure 7:**
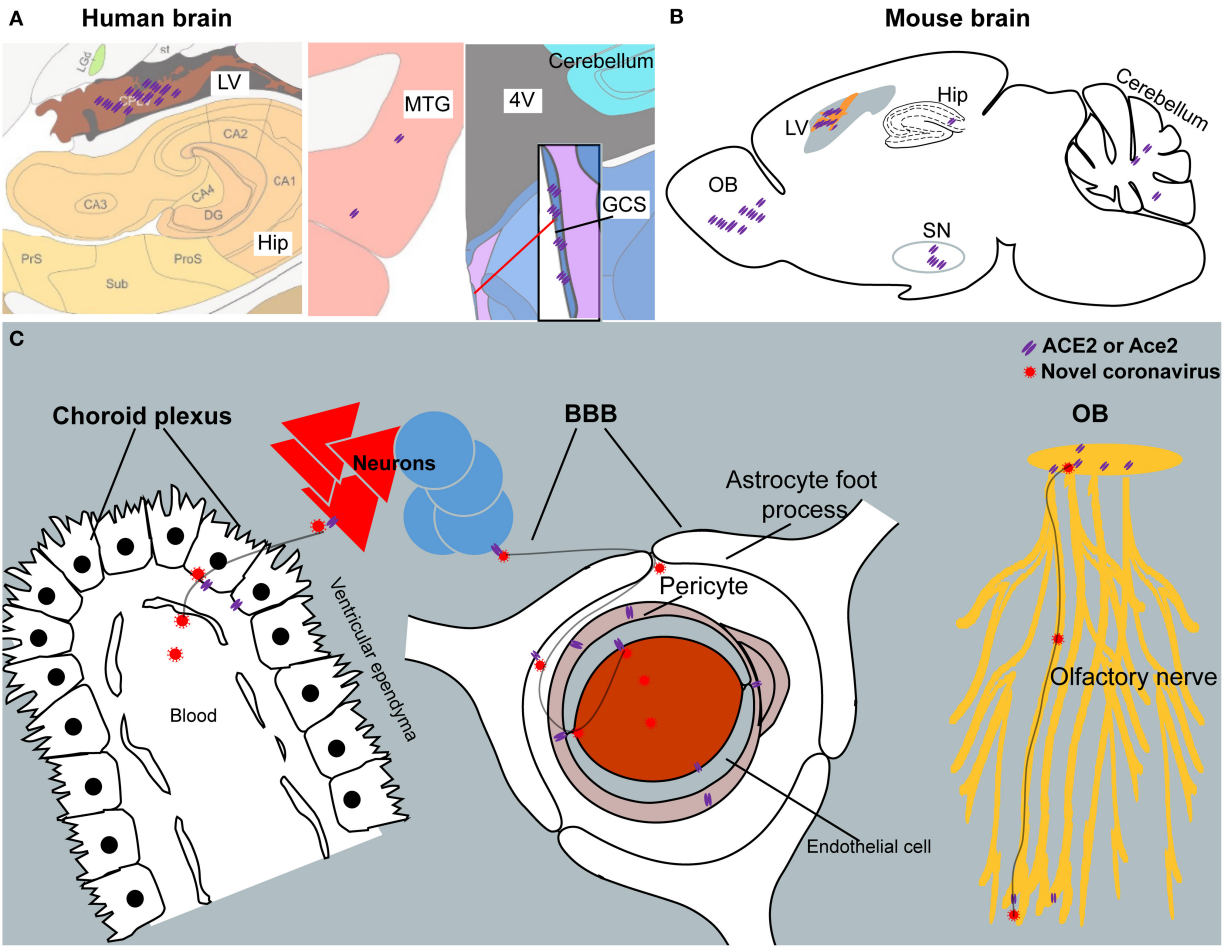
The summary schematic figure. **(A)** The distribution of ACE2 in the choroid plexus of the lateral ventricle (LV), hippocampus (Hip), middle temporal gyrus (MTG), and central glial substance (CGS) in the human brain. Pictures are modified from the human brain atlas (http://atlas.brain-map.org/) of the Allen Brain Atlas. **(B)** Representative ACE2 expression in the olfactory bulb (OB), choroid plexus of the lateral ventricle (LV), hippocampus (Hip), substantia nigra (SN), and cerebellum in the mouse brain. **(C)** Three potential routes for SARS-CoV-2 entering the CSF and/or spreading around the brain. Another potential route for SARS-CoV-2 entering the CSF from the CGS is not listed because of limited information regarding this brain area.

Our results reveal an outline of ACE2 or Ace2 distribution in the human and mouse brains, which indicates that the brain infection of SARS-CoV-2 might be capable to infect the brain and result in CNS symptoms in COVID-19 patients. The finding of high ACE2 expression in the central glial substance and brain ventricles suggests two potential novel routes for SARS-CoV-2 entering the CSF and/or spreading around the brain. A summary schematic figure is shown in Figure 7. In addition, the differences of ACE2/Ace2 distribution between humans and mice may be also useful to further “bench to bedside” translational studies regarding SARS-CoV-2. Our results may help to bring light to the brain infection of the present novel SARS-CoV-2 and previous SARS-CoV. Further studies are warranted to confirm our results and related predictions.

## Data Availability Statement

The original contributions presented in the study are included in the article/Supplementary Material, further inquiries can be directed to the corresponding author/s.

## Ethics Statement

Ethical review and approval was not required for the study on human participants in accordance with the local legislation and institutional requirements. Written informed consent for participation was not required for this study in accordance with the national legislation and the institutional requirements. Ethical review and approval was not required for the animal study because we just used publicly available brain transcriptome databases.

## Author Contributions

ZX and CW designed the study. RC, KW, and JY performed the search and analysis. DH and LF contributed to the data analysis for Figures 1, 2. ZX and KW checked the analyzed data. ZX wrote the manuscript in consultation with JY, ZC, CW, DH, and LF. All authors contributed to the article and approved the submitted version.

## Conflict of Interest

The authors declare that the research was conducted in the absence of any commercial or financial relationships that could be construed as a potential conflict of interest.
