# Supplementary material for: The Spatial and Cell-Type Distribution of SARS-CoV-2 Receptor ACE2 in the Human and Mouse Brains
Source: Front Neurol. 2021 Jan 20;11:573095. doi: 10.3389/fneur.2020.573095 (PMC7855591; doi:10.3389/fneur.2020.573095)
Supplement: Supplementary file 2 [file Data_Sheet_1.pdf]

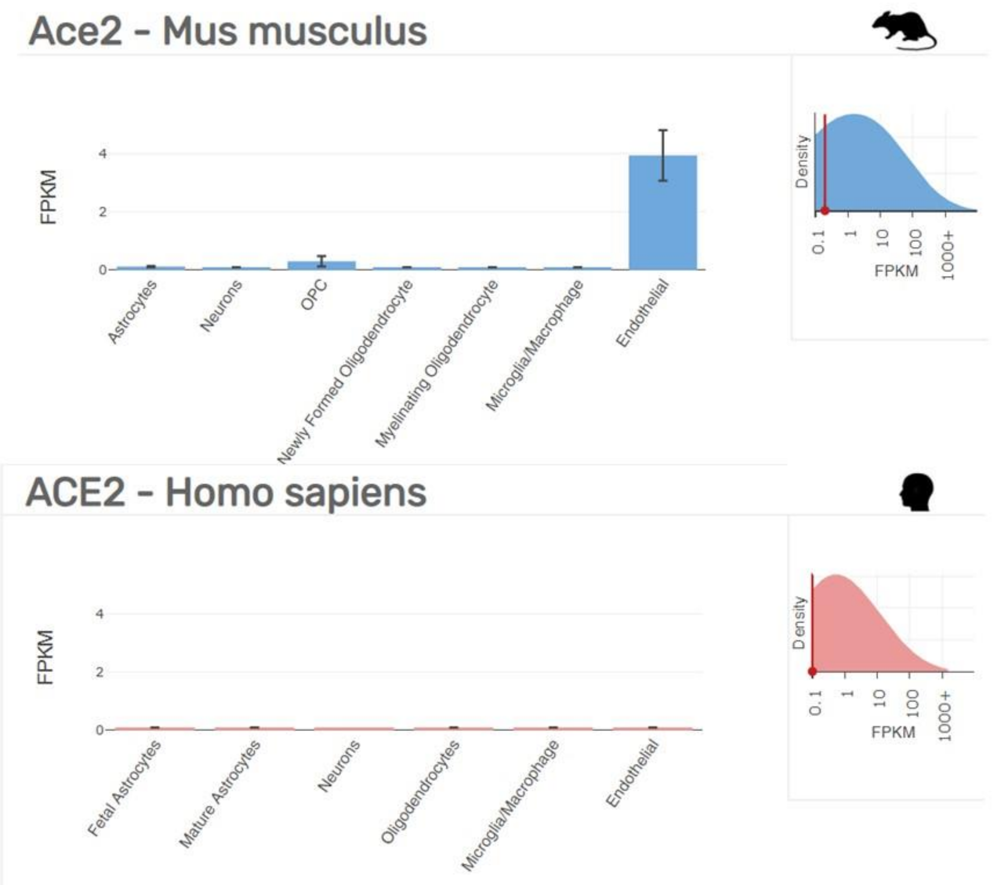

Supplementary Figure 1. Expression of ACE2 in the human and mouse according to Brain RNA-Seq database from Barres lab.

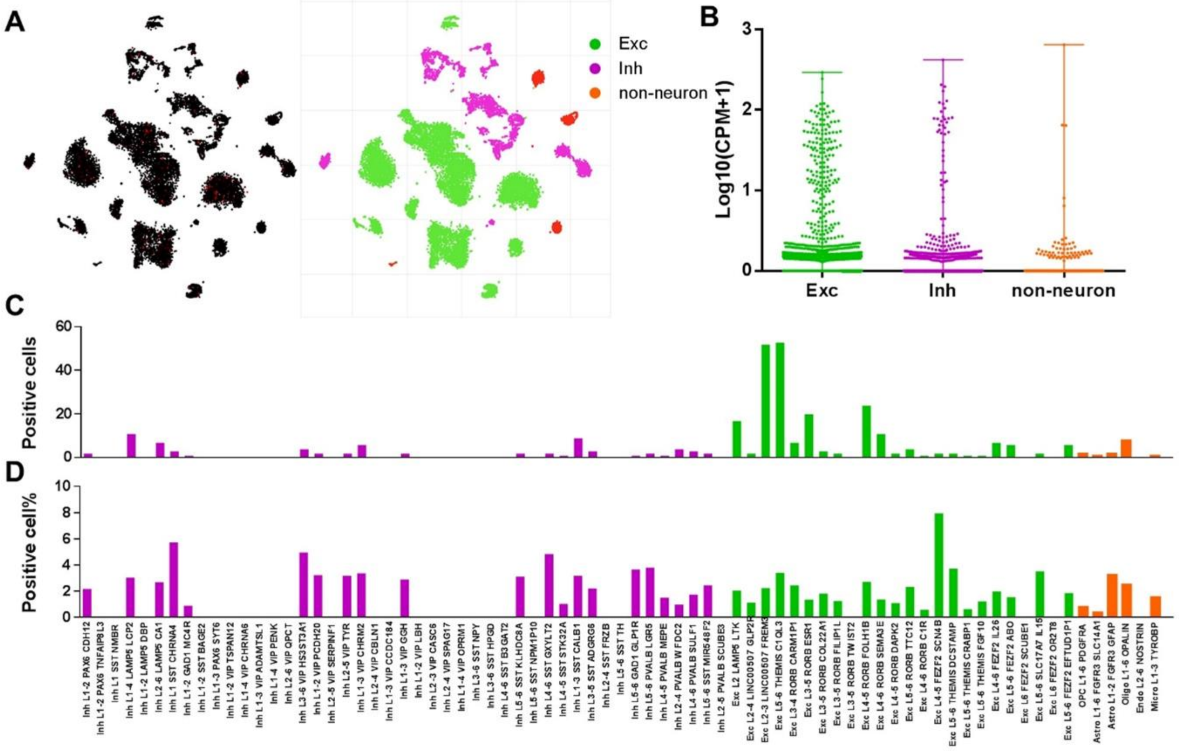

Supplementary Figure 2. ACE2 expression in the human middle temporal gyrus (related to Figure 4). (**A**) The expression of ACE2 in the cells of the human middle temporal gyrus. Left: Red means potential ACE2-positive cells, while black means ACE2-negative cells; Right: the annotation map of cell clusters. (**B**) The expression of ACE2 in brain cell subtypes. (**C**) The number of ACE2-positive cells in each sub-cluster of brain cells. (**D**) The percentage of ACE2-positive cells in each sub-cluster of brain cells. Cells with log10 (CPM+1) >0.3 were considered as positive cells. CPM: calculate counts per million; Inn: Inhibitory neuron; Exc: Excitatory neuron; Oligo: oligodendrocyte; Astro: astrocytes; Micro: microglia. Original data are from https://celltypes.brain-map.org/rnaseq/human/mtg. Images in A were directly generated by the RNA-Seq Data Navigator from Allen Cell Types Database (© 2015 Allen Institute for Brain Science. Allen Cell Types Database. Available from https://celltypes.brain-map.org/). Data are expressed as median, range, and all sample points in B.

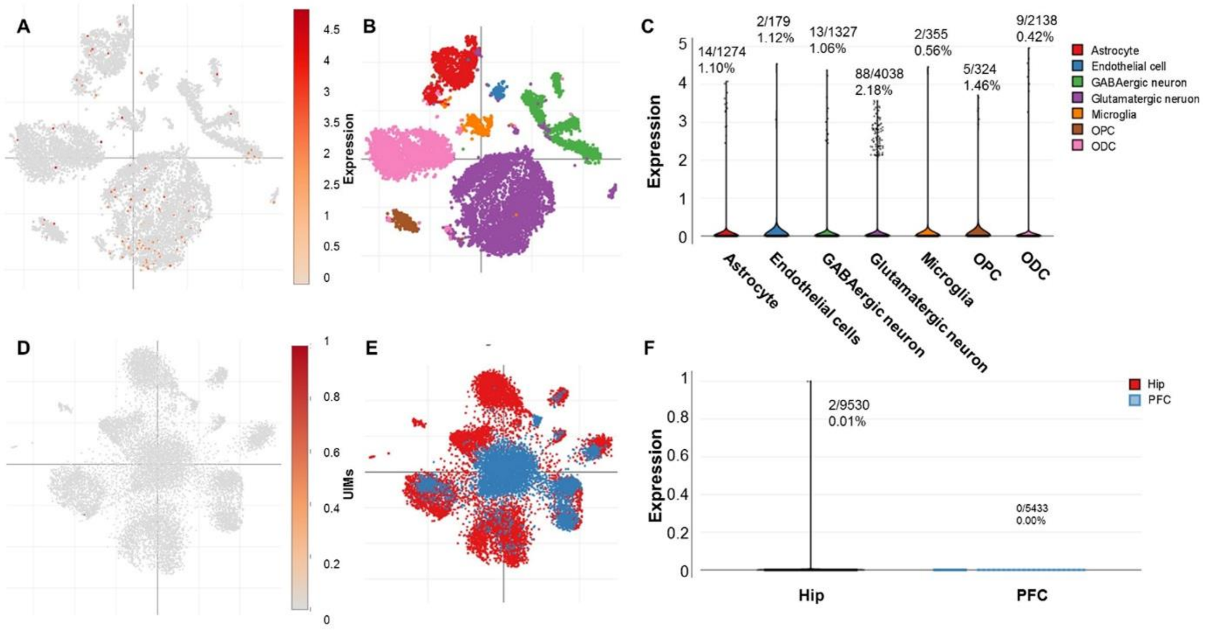

Supplementary Figure 3. Expression of ACE2 in the human posterior cingulate cortex, prefrontal cortex, and hippocampus (related to Figure 4). (**A**) Expression of ACE2 in the human posterior cingulate cortex. (**B**) The annotation map of cell clusters. (**C**) Expression of ACE2 in brain cell subtypes. (**D**) Expression of ACE2 in human prefrontal cortex and hippocampus. (**E**) The annotation map of brain regains, red points mean cells from the prefrontal cortex, and blue points mean cells from the hippocampus. (**F**) Expression of ACE2 in human prefrontal cortex and hippocampus. Original data are available from https://singlecell.broadinstitute.org. Data are expressed as median, range, and all sample points in C and F.

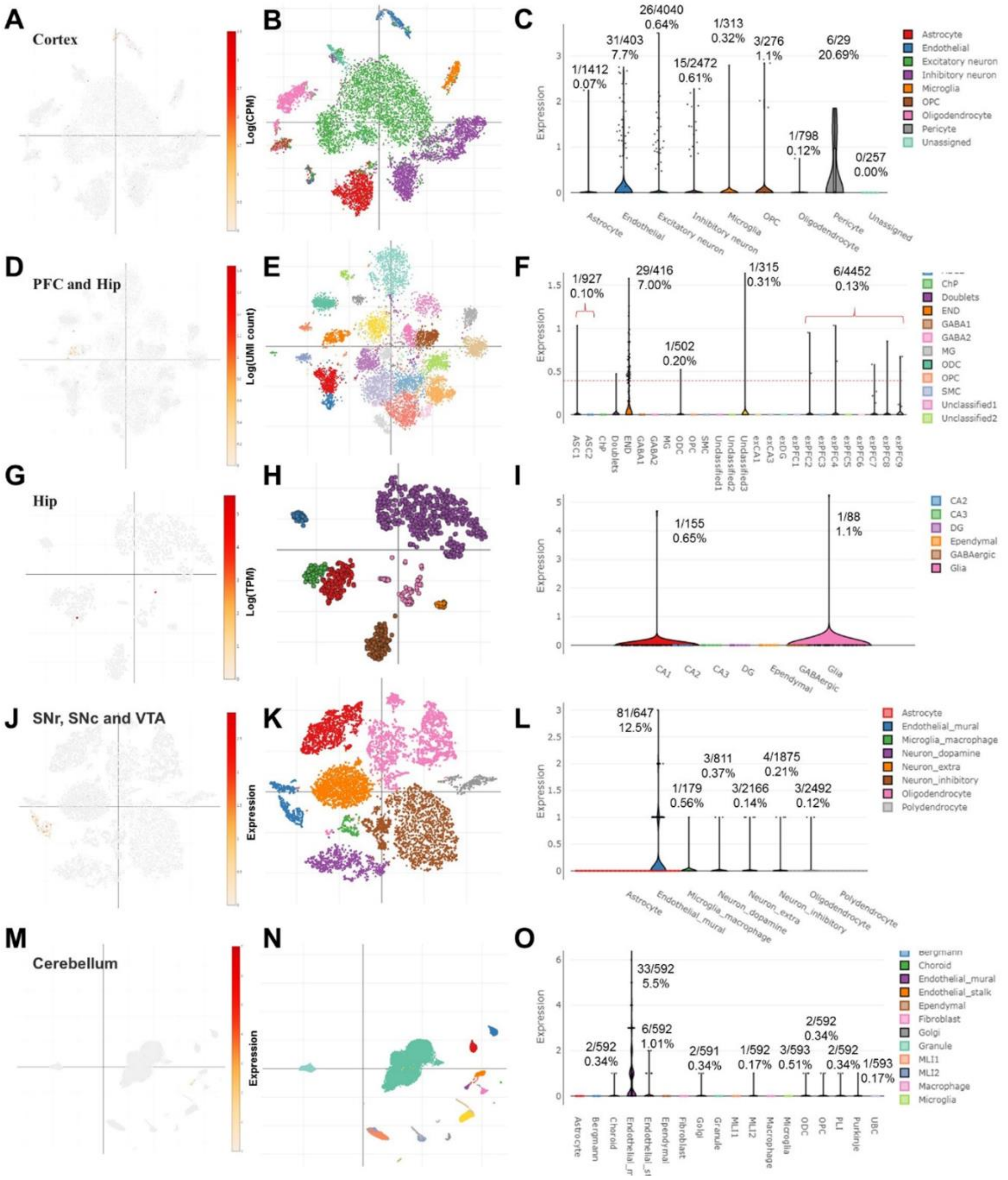

Supplementary Figure 4. Cell-type distribution of Ace2 in the mouse brain (related to figure 6). (**A-C**) Cell-type distribution of Ace2 in the mouse cortex. (**A**): Ace2 expression map; (**B**): the annotation map; (**C**) distribution plot. (**D-F**) Cell-type distribution of Ace2 in the mouse prefrontal cortex and hippocampus. (**D**): Ace2 expression map; (**E**): the annotation map; (**F**) distribution plot. (**G-I**) Cell-type distribution of Ace2 in mouse hippocampus. (**G**): Ace2 expression map; (**H**): the annotation map; (**I**) distribution plot. (**J-L**) Cell-type distribution of Ace2 in mouse SNr, SNc, and VTA. (**J**): expression map; (**K**): the annotation map; (**L**) distribution plot. (**M-O**) Cell-type distribution of Ace2 in mouse cerebellum. (**M**): Ace2 expression map; (**N**): the annotation map; (**O**) distribution plot. ACS: astrocyte; ChP: choroid plexus cell; END: endothelial cells; Hip: hippocampus; MG: microglia; MLI: molecular layer interneuron; ODC: oligodendrocyte; OPC: Oligodendrocyte progenitor cell. PFC: prefrontal cortex; SMC: smooth muscle cell; SNr: substantia nigra pars reticulate; SNc: substantia nigra pars compacta; VTA: ventral tegmental area; UBC: unipolar brush cells. Data are expressed as median, range and all sample points in **C, F, I, L, and O**. Original data are available from https://singlecell.broadinstitute.org.

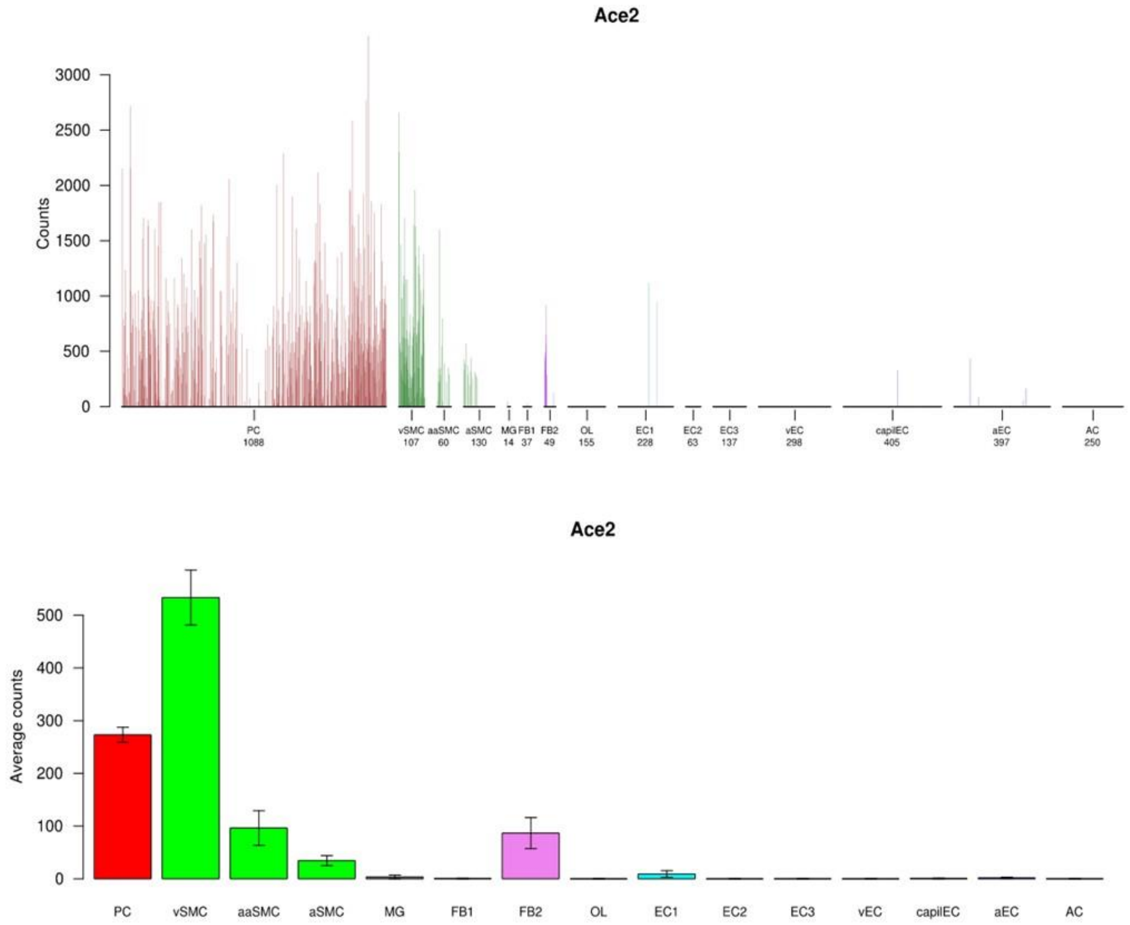

Supplementary Figure 5. Expression of ACE2 in mouse according to the vascular single-cell transcriptions database from Betsholtz lab. PC: Pericytes; SMC: Smooth muscle cells; MG: Microglia; FB: Vascular fibroblast-like cells; OL: Oligodendrocytes; EC: Endothelial cells; AC: Astrocytes; v: venous; capil: capillary; a: arterial; aa: arteriolar; 1,2,3: subtypes.

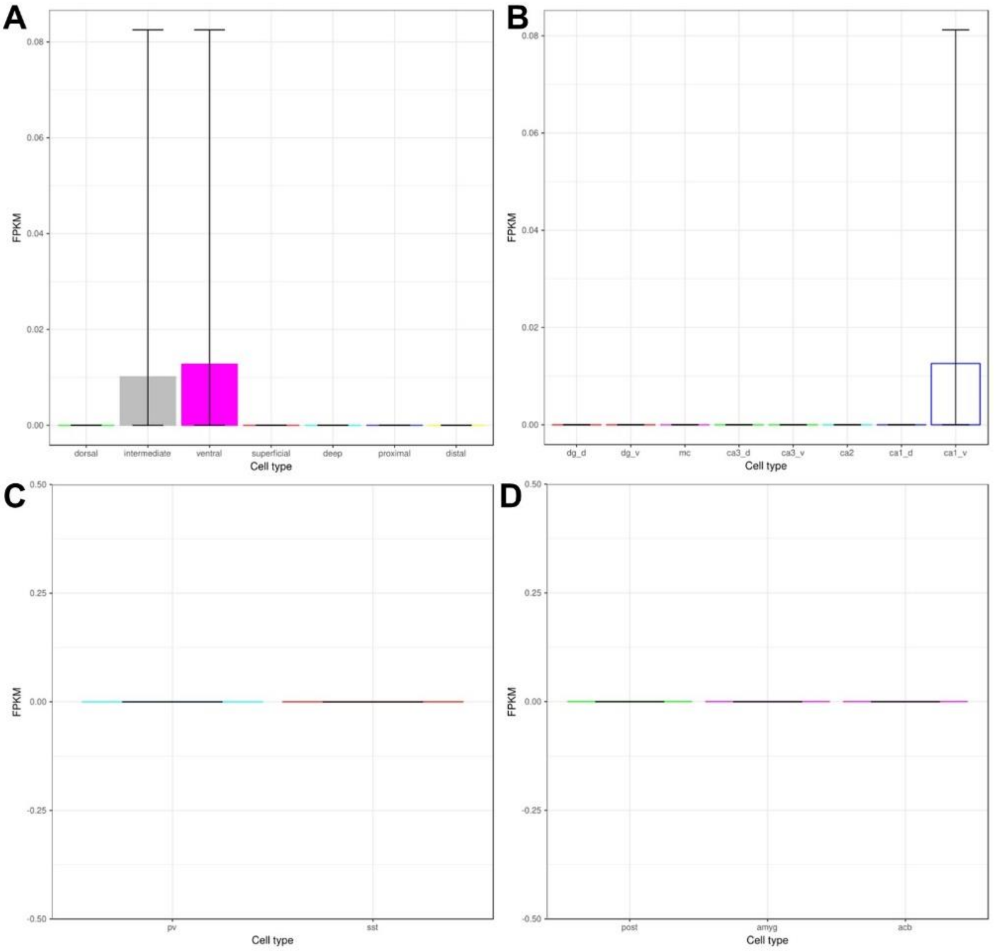

Supplementary Figure 6. Spatial and cell-type distribution of ACE2 in the mouse hippocampus (related to figure 6). (**A**) Spatial distribution of ACE2 in mouse hippocampus. (**B**) Spatial distribution of ACE2 in mouse hippocampal pyramidal cells and granule cells. dg_d: Dorsal DG granule cell; dg_v Ventral DG granule cell; mc: Dorsal DG mossy cell; ca3_d: Dorsal CA3 pyramidal cell; ca3_v: Ventral CA3 pyramidal cell; ca2: Dorsal CA2 pyramidal cell; ca1_d: Dorsal CA1 pyramidal cell; ca1_v: Ventral CA1 pyramidal cell (**C**) Cell-type distribution of ACE2 in mouse hippocampal interneurons. Pv: parvalbumin-expressing neurons; sst: somatostatin-expressing neurons. (**D**) Distribution of ACE2 in mouse hippocampal neurons that project to post-subiculum, nucleus accumbens or amygdala. post: post-subiculum projecting neuron; amyg: amygdala projecting neuron. NAc; nucleus accumbens projecting neuron. FPKM: Fragments Per Kilobase Million. Data are expressed as mean and 95%CI. Original data are available from: https://hipposeq.janelia.org (Cembrowski et al., 2016)
